# RT-QuIC and Related Assays for Detecting and Quantifying Prion-like Pathological Seeds of α-Synuclein

**DOI:** 10.3390/biom12040576

**Published:** 2022-04-14

**Authors:** Ankit Srivastava, Parvez Alam, Byron Caughey

**Affiliations:** Laboratory of Persistent Viral Diseases, Rocky Mountain Laboratories, National Institute of Allergy and Infectious Diseases, National Institutes of Health, Hamilton, MT 59840, USA; ankit.srivastava@nih.gov (A.S.); parvez.alam@nih.gov (P.A.)

**Keywords:** α-synuclein, prion, seed amplification assays, quantification, RT-QuIC, PMCA, Parkinson’s disease, Lewy body dementia, multiple system atrophy, synucleinopathies

## Abstract

Various disease-associated forms or strains of α-synuclein (αSyn^D^) can spread and accumulate in a prion-like fashion during synucleinopathies such as Parkinson’s disease (PD), Lewy body dementia (DLB), and multiple system atrophy (MSA). This capacity for self-propagation has enabled the development of seed amplification assays (SAAs) that can detect αSyn^D^ in clinical samples. Notably, α-synuclein real-time quaking-induced conversion (RT-QuIC) and protein misfolding cyclic amplification (PMCA) assays have evolved as ultrasensitive, specific, and relatively practical methods for detecting αSyn^D^ in a variety of biospecimens including brain tissue, CSF, skin, and olfactory mucosa from synucleinopathy patients. However, αSyn SAAs still lack concordance in detecting MSA and familial forms of PD/DLB, and the assay parameters show poor correlations with various clinical measures. End-point dilution analysis in αSyn RT-QuIC assays allows for the quantitation of relative amounts of αSyn^D^ seeding activity that may correlate moderately with clinical measures and levels of other biomarkers. Herein, we review recent advancements in α-synuclein SAAs for detecting αSyn^D^ and describe in detail the modified Spearman–Karber quantification algorithm used with end-point dilutions.

## 1. Introduction

Multiple neurodegenerative diseases (NDDs) are associated with accumulation of pathological aggregates of the protein α synuclein (αSyn). In Parkinson’s disease (PD) and dementia with Lewy bodies (DLB), disease-associated forms of αSyn (αSyn^D^) are major components of neuronal Lewy bodies (LB) and Lewy neurites, and in multiple system atrophy (MSA), αSyn^D^ accumulates in oligodendrocytes as glial cytoplasmic inclusions (GCIs) [1].

αSyn is normally a presynaptic neuronal protein that exists primarily as an intrinsically disordered monomer within the cytoplasm. However, in synucleinopathies, αSyn can be converted to β-sheet-rich, protease-resistant αSyn^D^ aggregates that grow by refolding and incorporating additional monomers [2,3,4]. Multiple studies have shown that αSyn^D^ can replicate and spread in a prion-like fashion within and between cells [5,6] and is considered the major culprit in the molecular pathology of synucleinopathies. The ‘dual-hit’ hypothesis postulates that an unknown trigger (e.g., an exogenous pathogen) is responsible for initiating the misfolding of native αSyn to yield assemblies that may then propagate via cellular communication mechanisms including passive diffusion, endocytosis, or exosomes [7]. Braak and colleagues described a pathological staging system in PD based on αSyn^D^ immunopositivity in the brain and other anatomical regions [8]. They proposed two plausible routes, i.e., nasal and gastric, for the spreading of αSyn^D^ and progression of PD.

This scenario may be analogous to the propagation mechanism(s) in prion diseases in which PrP^Sc^ (scrapie isoform of the prion protein) spreads between the tissues of infected hosts. Early evidence for prion-like spreading of αSyn aggregates was observed in the grafted dopaminergic neurons of patients who had undergone neuronal replacement therapy [9]. The presence of LB pathology within newly grafted neurons upon postmortem analysis supported the concept that αSyn^D^ can propagate between cells in the human brain. Also, cell-to-cell transfer of αSyn^D^ was observed in grafted neurons in a rat model [10].

The initiation and spread of LB pathology have also been observed using synthetic preformed αSyn fibrils (PFFs) in both cell and animal models [11,12]. Interestingly, PFFs convert into a GCI-like strain inside oligodendrocytes that maintains high seeding activity, even when propagated in neurons [13]. Furthermore, PFFs and other recombinant α-synuclein forms injected into the gastrointestinal tract of mice and rats induce LB-like aggregates in the brainstem via the vagus nerve [14,15,16]. Recently, Challis et al. observed that inoculating the duodenal wall of mice with PFFs led to changes in the enteric nervous system and gastrointestinal deficits [17]. Ultimately, inoculation of αSyn PFFs resulted in progression of αSyn histopathology to the midbrain in aged mice. Related, but not identical, pathological spreading has been seen in MSA, with αSyn^D^ having properties of infectious prions in cell cultures and animal models [18,19,20,21].

αSyn fibrils obtained from brain tissue or formed in vitro from recombinant protein [22,23,24] have been found to be structurally heterogenous (reviewed in [25]). Such αSyn fibril conformational polymorphs appear to be analogous to different prion strains and may be determinants of phenotypic diversity in synucleinopathies. Together, these observations support the *one disease, one strain* hypothesis for phenotypically distinct synucleinopathies [23,26]. To aid in synucleinopathy research and diagnostics, ultrasensitive seed amplification assays (SAAs) [27], including αSyn RT-QuIC [28,29] and the similar αSyn PMCA [30], have been developed to detect αSyn^D^ seeds in brain tissue, CSF, and other biospecimens. Herein, we review these assays and their utility in quantitating proteopathic seeds such as αSyn^D^ in biospecimens using an example dataset.

## 2. Diagnostic Potential of αSyn^D^

The clinical diagnosis of synucleinopathies and other NDDs has long been complicated by variable and overlapping symptoms, especially early in the pathologic process. Assessments of parkinsonism in patients can be helpful, but not definitive, with phenotypic manifestations including bradykinesia, resting tremor, rigidity, and postural instability. Parkinsonian traits are largely associated with PD and DLB and are less apparent in MSA [31]. Interestingly, DLB patients with parkinsonian traits are reportedly less responsive to L-DOPA (Levodopa) or similar treatments as compared with PD patients [32]. Another major difference is that DLB pathology involves a cognitive decline that is not found in PD patients, at least in the early and middle phases of disease. Thus, clinical diagnosis often involves a ‘one-year rule’, i.e., if cognitive alterations appear concurrent with, or before, movement symptoms, then the diagnosis is DLB and not late-phase PD dementia (PDD) [33].

It is also difficult to clinically differentiate synucleinopathies from other NDDs such as AD, progressive supranuclear palsy (PSP), corticobasal degeneration (CBD), and Creutzfeldt-Jakob disease (CJD) [34,35,36,37,38]. Even combined analyses of imaging (MRI, PET, and EMG) and fluid biomarkers (CSF Aβ42, NfL, *p*-tau, *t*-tau, αSyn, and HVA) do not routinely improve diagnostic accuracy for parkinsonism [39]. Thus, given the inconsistency and variability associated with current imaging and fluid biomarkers, the identification of more definitive biomarkers may help navigate these diagnostic ambiguities. Increasing evidence indicates that the detection of αSyn^D^ using SAAs can improve the clinical diagnosis of synucleinopathies [28,29,40,41,42,43,44], even in prodromal phases [27,40,45].

## 3. SAAs for Detecting Pathological Prion Aggregates

SAA platforms such as PMCA and RT-QuIC were initially developed to amplify, detect, and quantify pathological TSE (transmissible spongiform encephalopathy) prion aggregates in a variety of biospecimens. These assays were built on earlier observations that infectious prion protein aggregates, e.g., PrP^Sc^, induce the conversion of a normal cellular prion protein (PrP^C^) into an abnormal protease-resistant form in cell-free reactions with striking species and strain specificities [46,47,48,49,50,51]. PMCA reactions showed that such seeded conformational conversion occurs continuously under suitable conditions and is accompanied by increases in prion infectivity [27,52].

The original PMCA reactions involved the cyclic sonication and incubation of infected samples with vast excesses of normal brain homogenates containing PrP^C^ as the substrate for amplification [53]. PMCA products were then subjected to protease digestion and Western blotting to detect amplified conversion products. This protocol allowed the detection of prion aggregates with extraordinary sensitivity and selectivity [53,54]. However, limitations of this assay as a routine clinical test include the weeks-long reaction time for optimal sensitivity, the need for Western blotting, and the biohazardous infectivity of the amplified products.

**Figure 1:**
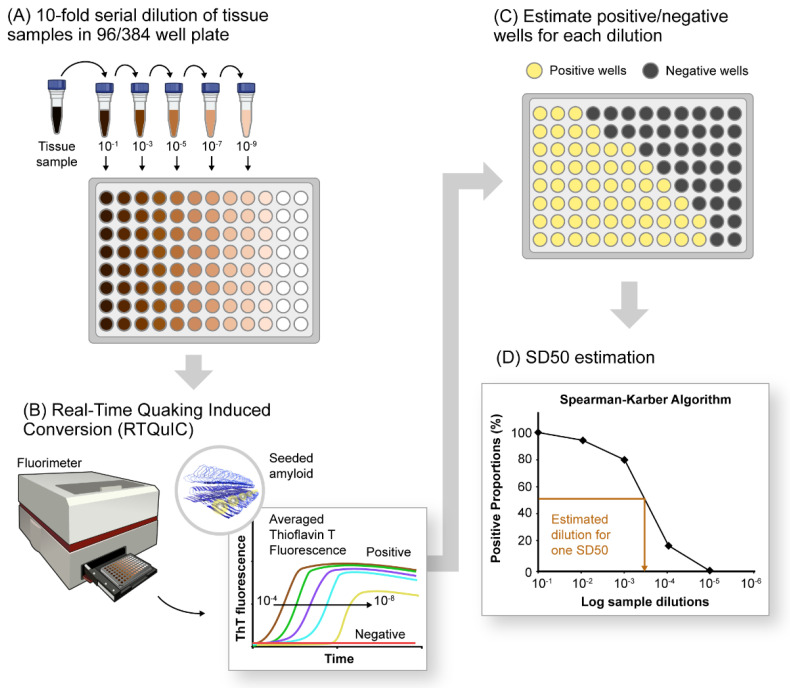
The modified Spearman–Karber method for relative quantification of proteopathic seeds in pathological tissues. (**A**) Serial dilutions (10−fold) of tissue sample (4 or 8 replicates) in a 96−well plate containing components of RT-QuIC reaction mixture including the recombinant, monomeric substrate protein and amyloid detection dye Thioflavin-T (ThT). (**B**) End-point estimation of serially diluted samples by the RT-QuIC assay. The resultant outcome is plotted as averaged ThT fluorescence versus time, showing declining fluorescence traces with increasing dilutions of positive samples. Negative samples do not exhibit any significant increase in ThT fluorescence under tested RT-QuIC conditions. (**C**) RT-QuIC outcomes for each dilution documented as number of positive and negative wells (for 4 or 8 replicates) per dilution tested. (**D**) Plot showing percentage of positive wells (shown as positive proportion percentages) for each sample dilution utilized for estimating seeding dose or sample dilutions in which 50% of the wells are ThT-positive (SD50).

The development of the amyloid seeding assay [55] and prion RT-QuIC [56,57] improved the practicality of SAAs by providing formats based on multiwell plates; shaking instead of sonication; recombinant, rather than brain-derived, PrP^C^ substrate; fluorescence (Thioflavin T (ThT)) instead of Western blot readout; much shorter assay times (e.g., 1–2 days); and noninfectious amplification products [58] (Figure 1A,B). Further development of prion RT-QuIC assays has improved their sensitivity and specificity and their applicability to most prion diseases (e.g., [59]); many specimen types including brain tissue [57,60,61], CSF [56,62,63,64,65], skin [66,67,68], eye [69], and olfactory mucosa [70,71,72]; and the cervid pregnancy microenvironment [73,74,75]. In some sample matrices, analytical sensitivities in the atto- or femtogram ranges have been documented [54,56,57,60,61,76,77,78,79].

## 4. SAAs for Detecting αSyn Aggregates

The biochemical framework of prion PMCA assays was later adapted for amplifying both αSyn PFFs and exogenous seeds present in tissue homogenates of transgenic αSyn mice [80,81]. Several αSyn SAAs are available for the detection of αSyn^D^ in various biospecimens. Among them, CSF is most commonly used for immediate as well as longitudinal diagnostics for most NDDs including synucleinopathies. In 2016, Fairfoul and colleagues first demonstrated RT-QuIC detection of αSyn^D^ seeding in a panel of 99 CSFs from DLB and PD patients with sensitivities of 92% and 95%, respectively, and 100% specificity against controls [28]. They observed lower RT-QuIC sensitivities in mixed pathologies including DLB with AD and AD with incidental LBs. A follow-up study reported 84% accuracy in discriminating α-synucleinopathies from non-synucleinopathies in a cohort of patients with parkinsonism of unclear etiology [42].

The closely related αSyn PMCA assay was described in 2017 [30]. This assay is similar in format to RT-QuIC, rather than the classical sonicated, brain-homogenate-based PMCA assays described above. A blinded analysis of a panel of PD and control CSF samples using a modified αSyn PMCA obtained an overall sensitivity of 88.5% and specificity of 96.9% [30]. This study also demonstrated 100% and 80% sensitivities for DLB and MSA CSF samples, respectively.

Thereafter, an improved and faster αSyn RT-QuIC assay (RT-QuICR) was reported by Groveman and colleagues that shortened the overall assay time to <2 days [29] as compared with 5–13 days for the earlier assays [28,30]. RT-QuICR utilizes a mutant K23Q recombinant αSyn substrate that is less prone to spontaneously fibrilizing than the wild-type substrate. RT-QuICR displayed a 93% diagnostic sensitivity for LB disorder (PD and DLB) CSF samples with 100% specificity against non-synucleinopathy and healthy controls. Intriguingly, the authors observed a difference in the average RT-QuIC fluorescence maxima obtained from PD and DLB CSFs, which they construed as possible strain differences analogous to those reported for different types of CJD cases in prion RT-QuIC reactions [59,64]. A similar RT-QuIC-based study detected αSyn seeds across a spectrum of LB-related disorders, including DLB and PD, with an overall sensitivity of 95.3% [82]. The use of a closely related αSyn RT-QuIC assay on a large panel of neuropathologically confirmed cases (*n* = 214) showed a 98% sensitivity for LB disorders and higher seeding activity in both brain homogenates and CSFs from DLB as compared with PD patients [83].

The above observations were corroborated by other studies showing faster RT-QuIC seeding kinetics for DLB compared with PD brain homogenates and CSFs [83,84]. A more recent RT-QuIC study showed that detergent-soluble fractions from PD frontal cortex had higher seeding efficiency compared with those from DLB frontal cortex [85]. Seeding differences in synucleinopathy samples were attributed to distinct αSyn fibril strains analogous to prion strains reported in other studies [22,24,86,87,88,89,90]. Altogether, αSyn SAAs provide protocols that require minimal hands-on time and increasingly rapid assay times for the accurate and ultrasensitive detection of αSyn^D^ seeds in PD- and DLB-derived brain tissue and CSFs.

## 5. Detection of αSyn^D^ Seeds in Peripheral Tissues

The spreading of αSyn^D^ to peripheral tissues has been reported for synucleinopathies both in patients and animal models [17,91,92]. αSyn^D^ was found in the lower gut, digestive system tissues, skin, and submandibular gland of clinically diagnosed synucleinopathy patients [93,94,95,96]. These findings suggest that peripheral tissues may represent diagnostic specimens that contain αSyn^D^ and can be collected using less-invasive means.

An RT-QuIC analysis of αSyn^D^ seeds in the submandibular gland from a small autopsied PD and incidental LB disease (ILBD) patient panel yielded 100% sensitivity [97]. The same group also found high RT-QuIC sensitivity using frozen skin tissue (96%) as compared with formaldehyde-fixed paraffin-embedded (FFPE) skin sections (75%) [98]. Another comparative study reported higher RT-QuIC sensitivity (93%) as compared with a sonicated PMCA assay (82%) in discriminating autopsied skin samples from clinically diagnosed PD patients and controls [99]. The authors replicated their results in skin biopsy samples from living PD patients by obtaining 95% sensitivity with RT-QuIC and 80% sensitivity with PMCA. Interestingly, the authors also saw higher RT-QuIC responses in posterior cervical tissues compared with leg skin tissues in live PD patients.

Feasibility of the use of skin tissue for PD diagnosis was further corroborated by another report of high RT-QuIC sensitivities with skin punches from both postmortem (88.9%) and live patients (89.3%) [100]. The authors found high concordance (96.2%) in a direct comparison between RT-QuIC outcomes for skin and CSF samples obtained from patients. The study also confirmed higher diagnostic efficacy for cervical skin tissue. More recently, a comparative two-center study obtained 92.2% concordance with a combined diagnostic accuracy of 88.9% for RT-QuIC performed on skin samples from multiple sites [101]. Their results also indicated better RT-QuIC accuracy from the combined analysis of cervical (C7) and thigh skin samples. Interestingly, significant correlations have been reported between the immunofluorescence of phosphorylated synuclein (PS-129) from skin nerves and RT-QuIC data from synucleinopathy patients [102].

Furthermore, RT-QuIC testing of OM brushings from PD and MSA patients showed sensitivities of 56% and 82%, respectively [44]. More recently, the comparative detection and quantification of αSyn^D^ seeds in PD samples across different postmortem tissues including the brain, skin, salivary gland, and colon were also assessed [83]. Overall, RT-QuIC-based testing for αSyn^D^ seeds in various peripheral tissues provides a less clinically invasive approach to diagnosis, albeit one that needs further validation.

## 6. Prodromal and Early-Stage Detection of αSyn^D^ Seeds by RT-QuIC

Idiopathic rapid-eye-movement (REM) sleep behavior disorder (iRBD) has significant co-occurrence frequencies in PD (15–65%), DLB (68–80%), and MSA (60–90%) patients [103,104,105,106]. iRBD is a parasomnia disorder with parkinsonian signs, cognitive dysfunction, and dopaminergic abnormalities [107,108,109]. Similarly, pure autonomic failure (PAF) involves the dysregulation of the autonomic nervous system with αSyn^D^ deposition in peripheral autonomic neurons [110,111]. Both iRBD and PAF are considered prodromal signatures, and their early detection helps in predicting the clinical onset of synucleinopathies and formulating clinical interventions [112,113].

Fairfoul and colleagues first reported positive RT-QuIC outcomes in CSFs from at-risk PD patients with the iRBD phenotype [28]. Another large patient cohort study of CSF demonstrated RT-QuIC sensitivities for iRBD and PAF patients of 100% and 92.9%, respectively [82]. More recently, a longitudinal CSF-based study showed 90.4% RT-QuIC sensitivity with 90% specificity for iRBD patients versus healthy controls [40]. The same study showed that 62% of the iRBD patients with positive RT-QuIC outcomes were diagnosed with PD or DLB within an average of 3.4 years. Interestingly, individuals with negative RT-QuIC outcomes showed a lower risk of developing synucleinopathy between 2–10 years after lumbar puncture. The diagnostic value of RT-QuIC in early-disease cases was validated by another large cohort CSF study (*n* = 289) that showed 97% sensitivity in detecting patients with mild cognitive impairment (MCI) due to probable Lewy body (LB) disease [45]. The utility of OM samples was also shown in a study reporting RT-QuIC sensitivities of 44.4% and 46.3% for iRBD and PD patients, respectively [114].

Another report on the simultaneous testing of CSFs and OMs from a blinded patient cohort demonstrated 81.4% sensitivity and 92.1% specificity in identifying probable or prodromal DLB cases [115]. For the most part, the above reports have indicated clinical utility of RT-QuIC assays in detecting probable, early-stage, and prodromal synucleinopathies. Importantly, from a practical perspective, multilaboratory collaborative studies have reported excellent concordance and high diagnostic performance in the use of αSyn SAAs (86%–96% sensitivity and 93%–100% specificity) for progressed as well as early-stage and prodromal PD cases [27,116].

## 7. Detecting αSyn^D^ Seeds in MSA Biospecimens

Versions of the αSyn PMCA assay have been shown to have a respectable 80% sensitivity for MSA cases using CSF samples [30]. However, αSyn RT-QuIC assays have had comparatively limited success in detecting and discriminating MSA. For example, Rossi and coworkers detected only 2 positive samples out of 31 clinically diagnosed MSA cases (6.5% sensitivity), while their two neuropathologically confirmed MSA samples remained RT-QuIC negative [82]. Similarly, van Rumund and colleagues achieved only 35% sensitivity for MSA, which was significantly lower than the value for their PD panel (84% sensitivity) [42]. Another recent study found only 4.4% RT-QuIC sensitivity in an MSA CSF patient panel as compared with 91.4% for CSFs from PD patients in the same cohort (*n* = 153) [117].

In contrast, an OM brushings study reported higher RT-QuIC sensitivity for MSA (82%) as compared with PD (56%) [44]. The authors also showed differences in proteinase-K resistance and electron-microscopy-based structural differences in RT-QuIC-amplified products, suggesting conformational strain differences in MSA and PD patients. These differences were further corroborated by an αSyn PMCA analysis that discriminated αSyn^D^ seeds in CSFs from PD and MSA patients with 95% sensitivity [118]. This report also showed evidence of structural differences between PD- and MSA-amplified aggregates using circular dichroism, FTIR, and cryo-electron tomography. The noted variable αSyn SAA sensitivities were possibly due to subtle differences in assay conditions or constituents but may also be attributed to clinical or genetic variability in MSA cases [87].

Genetic variability in MSA is not yet fully understood, and most cases are considered sporadic, with no familial history. On the other hand, biomarker variations associated with MSA biospecimens have been reported [119,120]. In fact, one study found high NfL concentrations in RT-QuIC-negative MSA CSF samples [42]. This was substantiated in another report that found markedly elevated NfL concentrations in both CSF (cNfL) and plasma (pNfL) from MSA patients that were RT-QuIC negative [117]. A combined assessment of cNfL and pNfL with RT-QuIC outcomes provided a higher diagnostic value (AUC 0.97) for discriminating PD and MSA patients.

Another major clinical variability is the presence of two different MSA phenotypes, i.e., the parkinsonian type (MSA-P), primarily characterized by parkinsonian features (slow movement, stiffness, and tremors), and the cerebellar type (MSA-C), with primary symptoms featuring ataxia, swallowing difficulties, and abnormal eye movements. A recent comparative assessment of OMs could only detect MSA-P with high sensitivity (90%), while most MSA-C samples remained RT-QuIC negative [83]. As suggested already by αSyn PMCA conditions [30,118], the development of SAAs with higher sensitivity for MSA and its subtypes may improve detection and discrimination in multiple clinical tissues.

## 8. Detection of αSyn^D^ in Familial Variants of Synucleinopathy

Familial PD cases represent a small proportion of PD patients that have genetic mutations that show either autosomal dominant (LRRK2 or SNCA gene) or autosomal recessive (PARK7, PINK1, or PRKN gene) inheritance patterns. The occurrence of certain atypical phenotypes and absence of LB pathology in major monogenic forms differentiates familial PD from classical PD (idiopathic, IPD) [121]. Similarly, genetic mutations in APOE, GBA, LRRK2, and SNCA genes have been shown to modulate risk for or cause DLB phenotypes [122]. To date, no definitive biomarker has been established for genetic variants of PD and DLB, presumably due, in part, to both genetic and pathological overlaps between them and with other NDDs, including AD [123].

Multiple CSF-based RT-QuIC and/or PMCA assessments have been reported for familial variants of synucleinopathies. A mixed-subjects cohort study showed that only 40% of LRRK2-PD individuals and 18.8% of LRRK2-NMC (nonmanifesting carrier) individuals had positive αSyn RT-QuIC responses [124]. Another CSF study reported RT-QuIC positivity only for PD and DLB patients carrying a GBA mutation, whereas LRRK2 carriers were RT-QuIC negative [82]. A later large cohort study on 236 PD and 49 DLB patients enriched for different mutations in GBA, parkin, PINK1, DJ1, and LRRK2 genes [125] found positive RT-QuIC outcomes only in patients carrying GBA (93%), LRRK2 (78%), or heterozygous mutations in recessive genes (59%), while all biallelic mutation carriers remained negative. Similarly, 100% of DLB patients carrying GBA mutations showed positive RT-QuIC outcomes in contrast with 79% of wild-type DLB patients. Patients with genetic PD variants (LRRK2 and GBA) included in another blinded CSF study did not show any differences in RT-QuIC or PMCA outcomes (97.1% concordant sensitivity) compared with patients lacking genetic variants of PD, and these results were concordant with clinical diagnoses [41]. Further study and SAA assay development are needed to better understand the impact of genetics on the detection of αSyn seeds in synucleinopathy patients.

## 9. Attempts to Correlate αSyn SAA Results with Clinical Measures

The diagnosis of synucleinopathies and other NDDs involves a number of clinical evaluations including the Unified Parkinson’s Disease Rating Scale-III motor scale (UPDRS-III), International Cerebellar Ataxia Rating Scale (ICARS), Mini-Mental State Examination score (MMSE), Montreal Cognitive Assessment (MoCA), Hoehn and Yahr (H and Y) scale, Schwab and England ADL (Activities of Daily Living) scale, Katz ADL scale, and Lawton ADL scale of disease severity, cognitive impairment, and disability [126]. While various RT-QuIC kinetic parameters, including lag phase (T_lag_), maximum ThT fluorescence (F_max_), time to F_max_ (T_50_), protein aggregation rate (PAR), and area under the curve (AUC), are utilized to discriminate synucleinopathy cases from controls, attempts to correlate these parameters with various clinical measures or evaluations have been less rewarding.

Rossi and coworkers showed a high F_max_ and AUC in LB-pathology-associated CSFs that discriminated them from non-LB pathologies such as MSA [82]. Similarly, other RT-QuIC studies reported the discrimination of synucleinopathies from controls in brain homogenate-, CSF-, OM-, and skin-tissue-based reports using the PAR parameter [44,83,97,98,127]. However, none of these studies found any correlation of these parameters with different clinical measures. Despite showing excellent predictive value in identifying PD cases (97.1% concordant sensitivity) in a larger patient cohort (BioFIND), both the F_max_ and T_50_ of SAAs (RT-QuIC or PMCA) failed to correlate with any clinical parameter [27]. Likewise, another large CSF panel study involving MCI-LB patients failed to find significant correlations between RT-QuIC kinetic parameters and several demographic and clinical evaluations [45].

Interestingly, in a follow-up study on the BioFIND CSF cohort with the RT-QuICR assay, weak correlations between F_max_ and clinical features such as motor deficit (MDS-UPDRS-III) and improvement in motor deficit with PD medications were noted for non-iRBD patients [116]. Also, probable-iRBD CSFs from the same study showed significantly shorter T_50_’s compared with those of non-iRBD samples. Previously, an αSyn PMCA study found a modest negative correlation of T_50_ with disease severity (H and Y) in two cohorts of PD patients tested in their study [30].

Furthermore, Kuzkina and colleagues’ study on PD skin samples utilized a unified skin RT-QuIC score defined as proportions of positive wells (for quadruplicate reactions; 1, 0.75, 0.5, 0.25, or 0) calculated for different biopsy sites and averaged for individual PD patients [101]. However, the unified score only showed modest correlations with clinical measures including disease duration, H and Y severity, MoCA score, and nonmotor symptoms (NMSs). Nonetheless, the unified scores were significantly higher in PD patients exhibiting prodromal or early-disease phenotypes including iRBD (0.93), constipation (0.94), and MCI (0.98). The unified scores also strongly correlated with the mean F_max_ for RT-QuIC assays taken together. However, the F_max_ showed weak correlations with both disease duration and disease severity (H and Y scale). Other kinetic parameters, including T_50_ and T_lag_, also displayed poor correlations with disease duration. Interestingly, the authors found that the quantitative SD50 parameter (described in the following section) was fourfold higher in patients with longer disease durations as compared with short duration patients. Similarly, a recent study on the Parkinson’s Progression Markers Initiative (PPMI) cohort reported that the RT-QuICR parameters F_max_, T_lag_, and AUC moderately correlated with disease duration and UPDRS-III scores [27]. The quantitative SD50 parameter also positively correlated with F_max_, age, and disease duration in the testing of CSF from PD subjects with advanced disease.

Overall, correlations of αSyn SAA parameters with various clinical measures have not been particularly impressive. Given that the seed concentration parameter, SD50/unit of sample, correlated slightly with age and/or disease duration parameters in two studies [27,101], it is possible that improved, more linearly responsive seed quantitation methodologies may allow for precise longitudinal assessments of synucleinopathies in a way that better correlates with clinical evaluations.

## 10. Quantification of αSyn^D^ Seeds Using RT-QuIC

End-point dilution RT-QuIC analyses have provided coarse-grained quantitative estimates of αSyn^D^ seed concentrations in biospecimens [29,116]. As was first done with prion RT-QuIC assays, such data can be analyzed via employing a modified Spearman–Karber method to compare seed concentrations by estimating the amount of samples containing enough seeding activity to yield positive (above-threshold) responses in 50% of technical replicate reactions. Analogous to median 50% lethal dose (LD50) estimations in animal bioassays of pathogens [128], the median seeding units showing 50% positive RT-QuIC reactions were designated SD50, for ‘50% seeding dose’ [57].

In this method, serial dilutions of a biospecimen are tested, and the proportion of positive (above-threshold) technical replicate reactions (the ‘positive proportion’) is tallied at each dilution (Figure 1A,C,D). The resulting data, ideally having at least one dilution yielding 100% positive replicate wells and another yielding 0%, are evaluated using a modified Spearman–Karber equation (Equation (1)) to estimate the log dilution of the original sample that, in the volume added to each reaction, contains an SD50 unit of seeding activity. Using this method, Groveman and colleagues compared seeding activities in brain tissue and CSF from PD and DLB patients. While the brain samples had 10^5^ to 10^6^ SD50 units per milligram of tissue, CSF samples only had 4 to 55 SD50 units per 15 µL. Similarly, other studies have utilized SD50 estimates to compare αSyn^D^ seed concentrations in diffuse neocortical DLB versus limbic DLB and in SMG versus brain tissues of PD patients [97,129].

**Figure 2:**
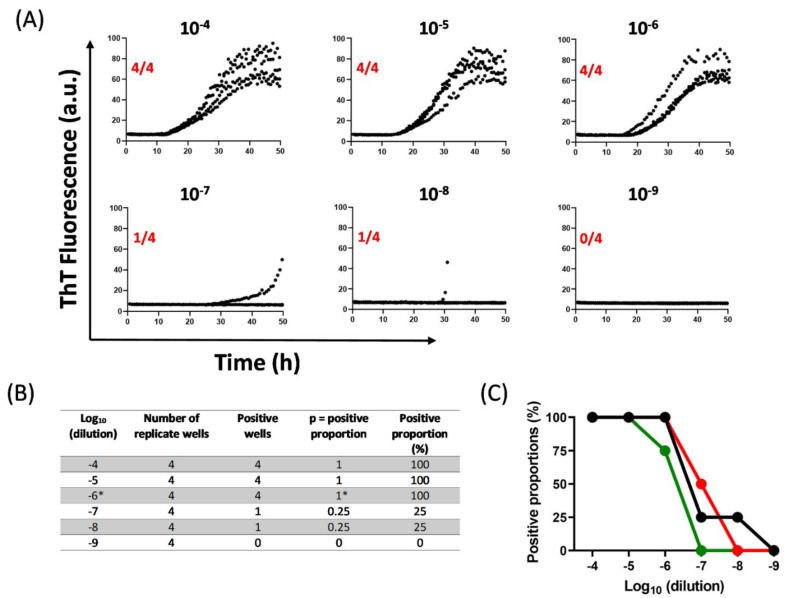
An example of modified Spearman–Karber analysis of brain tissues from a DLB patient (NIH NeuroBioBank) using the end-point estimations from RT-QuIC assay. (**A**) RT-QuIC outcomes for serially diluted (10-fold) brain tissue samples. The outcomes are representative of 4 replicates for each dilution shown as normalized ThT fluorescence signals (black traces) versus time in hours. Individual RT-QuIC outcomes are labelled for corresponding dilutions (in black) and positive outcomes per 4 replicates (in red). (**B**) Table showing positive proportions calculated for each dilution from an individual end-point dilution experiment. The ‘x’ value indicating the logarithm of the most extreme dilution at which 100% positive replicates (*p* = 1) is observed is highlighted with an asterisk. (**C**) Declining trends of positive outcomes per 4 replicates for each dilution (shown as positive proportions %) for three separate experiments are shown. Inset in the graph shows outcomes of similar end-point dilutions tested in RT-QuIC for a normal brain tissue (NIH NeuroBioBank). The resulting data are fitted in Equation (1), as shown below, to obtain SD50 estimates for the DLB brain tissue sample.

The algorithm for estimating SD50 from αSyn RT-QuIC assays is described in detail below using an example data set. For further clarification of Spearman–Karber mathematics, see https://www.cureffi.org/2015/09/20/the-math-behind-spearman-karber-analysis/ (accessed on 10 April 2022). In the provided example, RT-QuIC results for PD brain tissue dilutions (10^−4^ to 10^−9^) are first shown as ThT fluorescence in each of four technical replicate wells (Figure 2A).

Positive proportions (*p*) are then determined for each tested dilution (10^−4^ to 10^−9^; Figure 2B) and then applied to Equation (1):

log_10_ SD50 = x − d(S − 0.5) (1)

where: 

x = logarithm of the most extreme dilution at which 100% positive replicates (*p* = 1) is observed;

d = logarithm of fold dilution interval (i);

S = sum of positive proportions up to and including ‘x’.

For data shown in Figure 2:

x = log_10_ (10^−4^) = −6 (indicated with an asterisk in the table in Figure 2B);

d = log_10_ (10) = 1 (i =10 for a series of 10-fold dilutions);

S = 0.25 + 0.25 + 1= 1.5 (from table in Figure 2B).

Placing the above values in Equation (1):

log_10_ SD50 = −6 − 1(1.5 − 0.5) = −7

The resulting log_10_ SD50 represents the sample dilution at which the tested sample volume contains one SD50 of seeding activity. In this example, 10^−7^ is the estimated dilution at which the sample volume contains one SD50 unit of seeding activity.

With 1 µL of undiluted brain weighing ~1 mg, the amount of brain tissue in 1 µL of a 10^−7^ dilution = 1 × 10^−7^ mg brain. In this assay, 2 µL of each dilution was added to each well, so there was 1 SD50 per 2 × 10^−7^ mg brain tissue, or, 5 × 10^6^ SD50/mg brain.

Similarly, the described modified Spearman–Karber algorithm can be applied to any specimen, dilution factor, and volume of sample dilution added to each reaction. Finally, we found that although the approach described above (using 10-fold serial dilutions) yields quantitative discriminations of large differences in seed concentration on a logarithmic scale, it is only accurate, and reproducible, from +/−2- to 5-fold, at best, between independent assays of the same sample [57]. This reproducibility depends in part on the nature of the sample.

## 11. Conclusions and Perspectives

In summary, αSyn RT-QuIC and PMCA (SAA) assays exploit the self-propagation (seeding) activity of αSyn^D^ to allow detection in biospecimens. αSyn SAAs provide unprecedented diagnostic sensitivity and specificity for α-synucleinopathies using a variety of biospecimens including brain tissue, CSF, skin, nasal brushings, and other biological samples from synucleinopathy patients. Thus, αSyn^D^ seeding activity has become a clinically relevant biomarker for LB disorders, even in early prodromal synucleinopathy stages such as iRBD and PAF. Optimal detection of MSA αSyn^D^ requires assay conditions that are somewhat distinct from those more commonly used in αSyn RT-QuIC assays for LB disorders. Presumably, this is a consequence of MSA having a distinct conformer or strain of αSyn^D^. Recently reported SAAs have shown potential in discriminating different synucleinopathy strains as well as detecting multiple strains within the same subtype of disease [118,130]. These new insights may help in developing better SAAs for discriminating familial forms of synucleinopathies.

So far, correlations between αSyn SAA results and clinical measures that provide crucial information on disease progression and prognosis are weak or not significant. Such correlations might improve with the development of more consistent, precise, and linearly responsive αSyn^D^ seed quantification methods. Presumably, more practical methods that facilitate the use of higher replicate numbers and more narrowly spaced serial dilution increments will enhance statistical power, consistency, and accuracy. This, in turn, might improve the diagnostic and prognostic values of αSyn SAAs as well as their value in longitudinal assessments of synucleinopathy progression and therapeutics.
